# Health equity, care access and quality in headache – part 2

**DOI:** 10.1186/s10194-023-01699-7

**Published:** 2023-12-13

**Authors:** Bianca Raffaelli, Eloísa Rubio-Beltrán, Soo-Jin Cho, Roberto De Icco, Alejandro Labastida-Ramirez, Dilara Onan, Raffaele Ornello, Ruth Ruscheweyh, Marta Waliszewska-Prosół, Roberta Messina, Francesca Puledda

**Affiliations:** 1 Department of Neurology, Charité – Universitätsmedizin Berlin, corporate member of Freie Universität Berlin and Humboldt Universität Zu Berlin, Charitéplatz 1, 10117 Berlin, Germany; 2 Clinician Scientist Program, Berlin Institute of Health (BIH), Berlin, Germany; 3 Headache Group, Wolfson SPaRC, Institute of Psychiatry, Psychology and Neuroscience, King’s College London, London, UK; 4 Department of Neurology, Dongtan Sacred Heart Hospital, Hallym University College of Medicine, Hwaseong, Korea; 5 Department of Brain and Behavioral Sciences, University of Pavia, Pavia, Italy; 6 Headache Science & Neurorehabilitation Unit, IRCCS Mondino Foundation, Pavia, Italy; 7 Department of Physical Therapy and Rehabilitation, Faculty of Health Sciences, Yozgat Bozok University, Yozgat, Türkiye; 8 Department of Biotechnological and Applied Clinical Sciences, University of L’Aquila, L’Aquila, Italy; 9 Department of Neurology, LMU University Hospital, LMU Munich, Munich, Germany; 10 German Migraine and Headache Society, Frankfurt, Germany; 11 Department of Neurology, Wroclaw Medical University, Wroclaw, Poland; 12 Neuroimaging Research Unit and Neurology Unit, IRCCS San Raffaele Scientific Institute, Milan, Italy

**Keywords:** Health inequity, Worldwide health, Anti-CGRP drugs, Migraine, Cluster headache, Medication overuse

## Abstract

**Background:**

Headache disorders are a global public health concern affecting diverse populations. This review examines headache service organizations in low-, middle-, and high-income countries. It addresses global challenges in pharmacological headache treatment, with a focus on safety, tolerability, reproductive and child health, and outlines disparities in accessing innovative treatments worldwide.

**Main body:**

Organized headache services are essential due to the wide prevalence and varying severity of headache disorders. The tiered headache service model is globally recognized, although its implementation varies based on financial and workforce considerations. Headache burden affects well-being, causing disability, economic challenges, and work limitations, irrespective of location or income. All nations still require improved diagnosis and treatment, and the majority of countries face obstacles including limited access, awareness, economic barriers, and inadequate health policies. Provided adequate internet availability, telemedicine could help improve health equity by expanding access to headache care, since it can offer patients access to services without lengthy waiting times or extensive travel and can provide healthcare unavailable in underserved areas due to staff shortages.

Numerous health disparities restrict global access to many headache medications, especially impacting individuals historically excluded from randomized controlled trials, such as those with cardiovascular and cerebrovascular conditions, as well as pregnant women. Furthermore, despite advancements in researching migraine treatments for young patients, the options for treatment remain limited.

Access to headache treatment relies on factors like medication availability, approval, financial coverage, and healthcare provider expertise. Inadequate public awareness leads to neglect by policymakers and undertreatment by patients and healthcare providers. Global access discrepancies are exacerbated by the introduction of novel disease-specific medications, particularly impacting Asian, African, and Latin American nations excluded from clinical trials. While North America and Europe experience broad availability of migraine treatments, the majority of countries worldwide lack access to these therapies.

**Conclusions:**

Healthcare disparities, treatment access, and medication availability are concerning issues in headache medicine. Variations in national healthcare systems impact headache management, and costly innovative drugs are widening these gaps. Healthcare practitioners and experts should acknowledge these challenges and work towards minimizing access barriers for equitable global headache care in the future.

## Introduction

Headache disorders have long been a global public health concern, impacting individuals of all ages and backgrounds. Addressing the complex interplay of health equity, care access, and quality within the realm of headache medicine is pivotal to ensuring comprehensive and effective healthcare delivery to patients with headache.

**Fig. 1:**
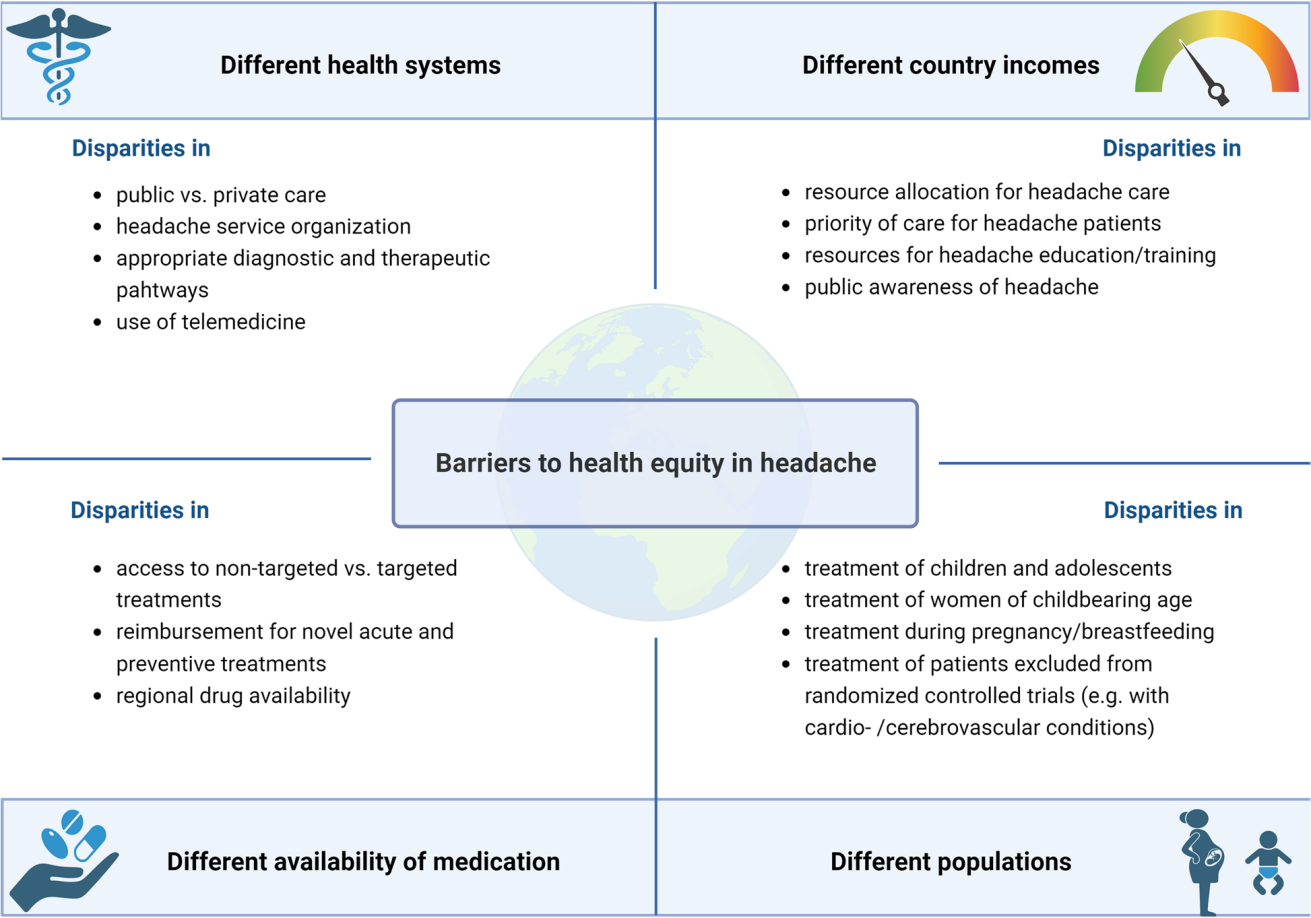
Barriers to health equity in headache addressed in this review. Figure created with BioRender.com

Building on Part 1, this review encompasses an exploration of headache service organizations, highlighting the hurdles and challenges in low-, middle- and high-income countries. Additionally, it delves into the challenges of pharmacological headache treatment from a global perspective, with a focus on safety and tolerability but also on reproductive and child health. Finally, it provides an overview of inequity in access to novel treatments around the world (Fig. 1).

The disparities in access, resources, and treatment options highlighted in this review underscore the pressing need for collaborative efforts aimed at achieving health equity for all individuals impacted by headache disorders. The diverse range of challenges and opportunities explored in this review is not intended to serve as an exhaustive overview of these topics. Instead, it is our aspiration that this review serves as a catalyst, sparking new discussions, insights, and ideas among patients, researchers, practitioners, policymakers, and stakeholders in the field.

### Headache service organization and health disparities

As headache disorders are highly prevalent in the population and can present with a broad spectrum of severity and disability, there is a need to create headache services that can stratify headache care [1]. An international consensus suggests a layered structure for headache services [2]. The first layer of headache care is represented by primary care settings managing most patients with headaches. Provided adequate resources, this first layer could meet up to 90% of the needs of headache patients. A second level of care includes neurologists and other headache physicians providing more specialized care for headache disorders. The third level of care includes highly specialized clinics providing advanced and multidisciplinary care for a minority of highly disabled patients with headache [2].

The multi-layered model of headache care has been applied differently across countries and health systems, depending on factors such as financial outlays or the availability of human resources [2, 3]. Some countries have adopted a bottom-up approach in which patients move from primary to specialized care, while others adopted a top-down approach with a focus on specialized care [2]. Moreover, in each country and system of care, there is a gradation of important and pressing problems (i.e., HIV, malaria, tuberculosis), resulting in a different distribution of resources and means for headache disorders [1].

In addition, health care in the vast majority of countries around the world is divided into public (free or insurance-funded) and private care [4]. Ideally, each of these care forms should assume the aforementioned three-tier care system. Unfortunately, public systems are most often underfunded, causing a lack of standard of care for headache patients, with staff lacking adequate education and limited access to highly specialized centers [2]. Private care generally provides more expensive but better quality of care due to the wide availability of resources, however, the standards of care and expertise are not always consistent [2].

#### Challenges in low- and middle-income countries

Headache burden significantly and greatly affects the health of individuals, causing disability, economic and labor losses, regardless of geography and income [5]. In low- and middle-income countries, insufficient resources are major challenges for both health professionals and patients [1]. Individuals with headache disorders often try to cover the disease costs themselves, which widens the social gap between those who can afford it and those who cannot [6].

For example, in India, basic migraine treatments can be offered free of charge in public and private centers. However, due to India's large population, inadequate care facilities and access difficulties from rural areas, public centers cannot provide adequate care for every patient. Moreover, migraine is still not considered a neurological disease, limiting coverage by health insurance [7–9].

In China, almost 33% of patients with migraine are misdiagnosed, and almost half of them are not diagnosed at all. However, since the 2010s, 135 headache clinics have been established, guidelines have been prepared, and progress has been made in the management of migraine [10, 11].

In Latin American countries, insufficient headache care resources in public institutions often lead to misdiagnoses and inadequate treatment. Although the diagnosis and treatment methods are better in private institutions, only some patients can afford care services financially [12]. Moreover, specific headache education for health professionals is lacking [13].

In Russia, there are growing efforts to increase migraine awareness with the cooperation of public centers and the Ministry of Health. Tertiary headache centers for the diagnosis and treatment of migraine in public centers have been implemented in more than 30 cities. In addition, the training of health professionals is being improved, for instance, by translating the ICHD criteria into Russian language [1].

Türkiye has free public hospitals, university hospitals, and private hospitals as centers for headache treatment. However, long waiting times can be challenging for patients in the public sector [14].

Taking these examples together, challenges in low- and middle-income countries include: access to care, lack of education and awareness for headache disorders, economic barriers in the diagnosis-treatment process, and inadequate health policies. Despite growing efforts to facilitate headache care in low- and middle-income countries, there is still a long way to go to guarantee proper diagnosis and treatment for these disorders worldwide.

#### Challenges in high-income countries

A fundamental problem in high-income countries is the low priority of care for headache patients. There are apparent educational deficiencies already at the level of medical studies, during which little time is devoted to headache education. As a result, inadequate diagnosis and treatment can greatly limit access to proper medical care in this group of patients, despite the existence of effective therapies [15]. The greatest burden on patients, as well as countries and societies, is caused by migraine, tension-type headache, and medication overuse headache (MOH) [5]. It appears that in countries with access to medical and pharmacological care, MOH can account for up to 50% of all headache types [1, 2].

One challenge for patients with headache seeking medical care is often the unclear diagnostic and therapeutic pathway. A patient with headache can easily get lost in the system and can end up being managed by the wrong specialists, such as neurosurgeons, ENT surgeons, dentists, or ophthalmologists [16]. For example, only one in four patients with headache in Luxembourg, one in three in Spain, and one in five in Greece reported seeing a neurologist, most of them privately [4, 17]. In Denmark, almost 25% of patients have never consulted a healthcare professional for headache, even though they consider headache a clear burden in their daily lives [18]. This percentage is much higher in countries like Poland, with almost 94% of patients who have consulted a physician highlighting possible societal and cultural differences towards headache disorders [19]. In the US, the percentage of specialist consultations has increased over the years, from 16% in 1984 to almost 80% in 2018 [20, 21].

Despite the increasing percentage of patients consulting a physician about their headaches, proper diagnosis and implementation of appropriate treatment still needs to be addressed. In fact, the correct diagnosis of migraine can still be problematic [19, 22]. In terms of treatment, the biggest problem in high-income countries seems to be the proper use of prophylactic therapy, which, according to data from various countries, ranges from 10–20% of patients requiring such treatment [19, 23, 24]. In the context of prophylactic treatment with the latest and most effective drugs (monoclonal antibodies, gepants, botulinum toxin A), the problem remains the reimbursement of treatment, which depends on the financial policies of each country. These therapies are often reserved only for the most severely ill patients who have undergone multiple unsuccessful treatment attempts [25].

### Health disparities in telemedicine for headache

Telemedicine can be defined as the remote delivery of healthcare via telecommunication systems [26]. Telemedicine can allow patients access to services that would otherwise require long waiting time and long-distance travel. Additionally, it could enable access to health services that are not available in underserved populations. Therefore, telemedicine could substantially contribute to health equity by meeting the need for increased access to headache care [27]. On the other hand, enhancing telemedicine can generate further disparities, as health services need to invest in technologies and infrastructures to deliver high-quality services that also protect patients’ data. Moreover, patients accessing telemedicine should have adequate computer literacy, good cognitive status, and should have a good home infrastructure for receiving the service.

Besides providing better care to many patients, more expansive use of telemedicine could enhance access to services, such as behavioral treatments, that are currently underused mainly because of a shortage of dedicated professionals [28]. Several reports on the use of telemedicine in headache care showed that the quality of service and patient satisfaction were comparable to in-person services [29, 30]. Those reports include both randomized controlled trials (RCTs) [31, 32] and real-world studies [33–36]. However, it should be noted that those reports dealt with selected patients, and did not include an assessment of patients excluded from the services due to feasibility reasons or refusal to access telemedicine. Besides, the high prevalence of headache disorders in the general population implies a rational use of telemedicine resources for headache care [29]. There is the risk that patients who could access lower layers of headache care are brought to unnecessary access to higher layers because of increased accessibility of services via telemedicine, therefore leading to a saturation of the system [29]. In low- and middle-income countries, a further problem lies within the limited resources that can be allocated to telemedicine infrastructures and personnel training. Even in the most developed settings, there could be inequalities referring to underserved populations and ethnic minorities [30]. Relying on a diversified offer of telemedicine services that includes access via commonly used devices such as smartphones, is a viable option [37].

**Fig. 2:**
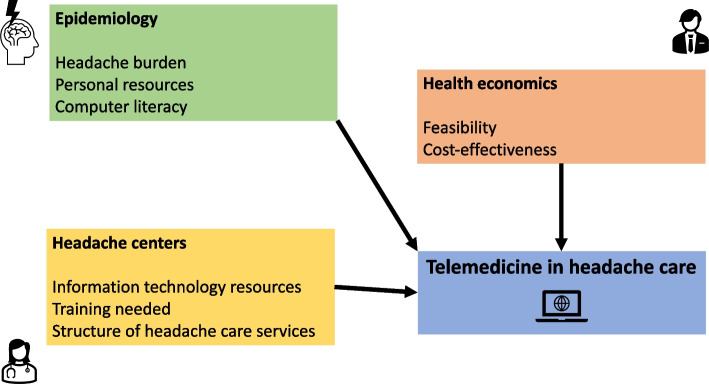
Factors affecting the implementation of telemedicine for headache care and related health disparities. A list of disciplines involved in considering those factors is provided

A rational use of telemedicine in low-, middle- and high-income countries aimed at preventing health disparities implies several considerations:

- obtaining precise epidemiological data on the prevalence and burden of headache disorders in the population of interest;
- estimating the number of subjects that could benefit from telemedicine approaches across the different levels of headache care, taking into account the computer literacy and access to electronic infrastructures of the population of interest;
- estimating the amount of resources needed to acquire infrastructures and train personnel;
- performing feasibility and cost-effectiveness analyses (Fig. 2).

To provide this service, collaborations among epidemiologists, public health experts, experts in health economics, headache care providers, information technology experts, and ideally patients’ representatives are advised.

### Safety and tolerability issues of headache medication from a global perspective

Multiple health disparities limit access to most headache medications worldwide. This is especially true for populations that have been historically, socially, and economically marginalized from biomedical research and treatment [38, 39]. This section will discuss the safety and tolerability problems of the current headache medications included in the World Health Organization (WHO) Model List of Essential Medicines [40]. These represent the minimum medicine needs for a basic and cost-effective healthcare system. Moreover, since healthcare systems and drug availability vary greatly among the different socio-economic and geographical areas, we will also discuss the most widely available antimigraine drugs, based on the results of the Atlas Of Headache Disorders and Resources in the World [39] as well as the most commonly prescribed novel medications approved for the treatment of headache.

The WHO core list includes three simple, non-opioid analgesics (acetylsalicylic acid, ibuprofen, paracetamol) as well as sumatriptan for the acute treatment of migraine attacks, whereas propranolol is the only therapy currently included for migraine prophylaxis. In general, several studies have shown that simple analgesics are a widely available and effective first-line treatment option for acute treatment of mild-to-moderate migraine attacks and tension-type headache attacks [39, 41–43]. Analgesics have favorable safety profiles, and although there have been some concerns over the safety of paracetamol in people with compromised hepatic function, these issues have not been confirmed at standard therapeutic dosage [42]. These therapies are also well-tolerated, and studies reported no serious adverse events [41–43]; unfortunately, many patients do not obtain adequate pain relief and often require combination analgesic medications or an analgesic plus a headache-specific acute treatment.

Sumatriptan, one of the most widely available specific antimigraine drugs according to the WHO [39], is the prototypical triptan, a 5-HT_1B/1D/(1F)_ receptor agonist, believed to exert its effect through modulation of the release of calcitonin gene-related peptide (CGRP) [44–46]. It is an effective abortive treatment for moderate-to-severe migraine attacks and cluster headache [47, 48]. Sumatriptan has proven to be safe for most patients without known cardiovascular disease [48, 49]; however, due to the possible vasoconstriction of arteries, it should be avoided in patients with atherosclerotic disease, such as coronary and peripheral artery disease [50, 51]. It is estimated that up to 40% of sumatriptan-treated participants experience at least one adverse event within 24 h of treatment, with most being of mild or moderate severity and self-limiting [48]. Similar considerations apply for all seven existing triptans [52], although their commercial availability and prices vary significantly from country to country. Finally, the regular intake of triptans and analgesics has additional tolerability challenges, as it increases the risk of developing adverse events and medication overuse headache, increasing the burden of headache patients [53]. In relation to this, it is estimated that in upper-middle- and high-income countries, 10% of specialist consultations are related to medication-overuse headache, compared to only 1% in low-income countries [39].

The most widely available specific anti-migraine drugs worldwide are ergot alkaloids [39], which are non-selective 5-HT receptor agonists with D_2_ and α-adrenoceptor affinity [45, 54]. Both ergotamine and dihydroergotamine have been on the market for over 70 years, but studies assessing their efficacy are limited [54]. In the last decade, novel pharmaceutical formulations of dihydroergotamine have been developed, with studies showing moderate efficacy [55, 56]. Due to the vast array of receptors ergots bind to, the risk of side effects is greater. In line with this, nausea, vomiting, weakness in the legs, muscle pain, numbness, tingling, edema, and bradycardia have all been reported, together with coronary vasoconstriction [51, 57]. Therefore, these drugs are also contraindicated in patients with cardiovascular disease. As a result, they have been removed from the list of essential medicines of the WHO, and the European Medicines Agency (EMA) recommended restricting their use since the risks are greater than the benefits [39, 58].

Propranolol, a non-selective beta-blocker, is one of the most commonly prescribed drugs for migraine prophylaxis and the most widely available prophylactic in the world [39, 59]. While propranolol is considered effective, safe, and well-tolerated in the short-term interval treatment of migraine [59], its blood pressure-lowering profile and non-selective properties are associated with a variety of adverse effects, such as bradycardia, hypotension, vertigo, bronchospasm and gastrointestinal complaints [59, 60]. Moreover, propranolol use may be limited in patients over 60 or with low baseline blood pressure, and when its use can exacerbate comorbid conditions such as asthma, peripheral vascular disease, and cardiac conduction disturbances [61]. Even in countries where other preventive drugs are available, tolerability issues often hamper treatment adherence [62, 63], challenging the long-term treatment of many patients worldwide.

In the last years, several novel drugs have been developed and approved for the treatment of migraine and cluster headache, which target directly or indirectly CGRP signaling. Lasmiditan, a lipophilic and selective 5-HT_1F_ receptor agonist, inhibits the release of CGRP from peripheral and central trigeminal terminals [44]. Multiple RCTs have demonstrated that lasmiditan has a good safety profile [64], with no apparent issues in patients with cardiovascular risk factors [65], matching its lack of vasoconstrictor activity [66]. However, due to its ability to cross the blood–brain barrier, patients report a high incidence of central nervous system-related adverse effects like dizziness, vertigo, and somnolence [67], which can impair daily activities.

The new generation of gepants, selective small-molecule CGRP receptor antagonists, have all proved to be effective in the acute (ubrogepant, rimegepant, and zavegepant) and preventive (atogepant and rimegepant) treatment of migraine. These drugs have shown safety profiles with no demonstrable abnormalities in liver function or pharmacokinetic issues, as seen with the first generation of gepants [68]. Moreover, they could be a more affordable option for monoclonal antibodies against CGRP or its receptor. Considering the role of CGRP in the maintenance of (cardio)vascular homeostasis in pathophysiological conditions, and the higher cardiovascular risk of migraine patients, blockade of this signaling pathway poses a concern [69]; therefore, it is crucial to consider preexisting cardiovascular risk factors in patients (i.e., family history, tobacco exposure, obesity) to prevent possible cardiovascular events.

It is evident that unless health inequities and limited access to novel headache medications are improved worldwide, underrepresented populations will continue suffering disproportionately. Additionally, healthcare education and long-term studies are highly needed to address the gaps in healthcare treatment, particularly in vulnerable populations.

### Reproductive health considerations in headache medicine

Migraine affects women two- to three times more often than men, with a prevalence peak during reproductive years [70]. Addressing women’s health needs throughout different life phases is essential for comprehensive migraine care. Key considerations involve the impact of sex hormones on the migraine course, the choice of contraception, family planning, pregnancy, and childbirth [71]. In particular, pregnancy and lactation can limit the use of many migraine medications, as they can pose risks to the developing fetus or the infant [72]. While most female patients report an improvement of migraine burden during pregnancy, migraine attacks still occur in a significant proportion of pregnant women that are not always manageable with non-pharmacological treatments only [73].

For acute treatment of migraine attacks during pregnancy, non-steroidal anti-inflammatory drugs (NSAIDs) can be administered during the first two trimesters. Their use in the third trimester is contraindicated, as they might lead to the premature closure of the ductus arteriosus [74–76]. Paracetamol was considered the medication of first choice during pregnancy for a long time. Recently, concerns have been raised regarding fetal neurological development, asthma, or testicular undescent in boys [77]. It still belongs to the recommended acute drugs during pregnancy, particularly during the third trimester, but its intake should be carefully pondered under risk–benefit assessment. For more severe attacks, triptans can be considered, as extensive clinical experience does not point to teratogenicity [78]. Paracetamol and NSAIDs are also regarded as compatible with breastfeeding [74]. Triptans should be used with caution during breastfeeding, mainly due to the lack of robust data [74]. However, the relative infant dose is very low, especially for eletriptan, suggesting high safety [74].

Regarding preventive drug treatment, it is even more important to weigh the potential benefits of the medication against its risk. Especially valproate and topiramate, two anticonvulsants commonly used as migraine preventive drugs, have proven teratogenic effects, and should be avoided [79–81]. Valproate use during pregnancy has been associated with a dose-dependent increased risk of congenital malformations, developmental delays, and cognitive impairment in exposed infants. Consequently, valproate should ideally not be used in women of childbearing potential. Similarly, topiramate has been associated with an increased risk of major congenital malformations, fetal loss, prenatal growth retardation, autism spectrum disorder, and intellectual disability [79, 80]. During breastfeeding, it is recommended to monitor newborns for signs of sedation like poor suckling, irritability, diarrhea and weight loss, since topiramate levels are estimated to reach up to 25% of maternal levels [74, 82].

Alternatives to antiepileptics include beta-blockers, antidepressants, and OnabotulinumtoxinA. In the case of beta-blockers, propranolol is considered a safe option during pregnancy and breastfeeding; however, it is important to consider that propranolol efficacy has been shown to vary depending on the hormonal status of patients [83], and when taken in the third trimester, it should be stopped a few days before delivery since there is an increased risk of respiratory depression, neonatal bradycardia, and hypoglycemia [73, 74, 84]. Tricyclic antidepressants like amitriptyline can also be an alternative, but they should be taken with caution since their intake during the third trimester could result in neonatal withdrawal symptoms. During lactation, they are considered a safe option, but it is recommended to monitor for anticholinergic symptoms like dry mouth or constipation [74, 84]. Lastly, onabotulinumtoxinA is not expected to enter the systemic circulation or to transfer to breast milk due to its high molecular weight, and retrospective studies have not observed an effect on pregnancy outcomes, but further studies are warranted to assess its safety [85, 86].

It is important to consider the possibility of an unplanned pregnancy in a patient under treatment with one of the antibodies targeting the CGRP pathway, especially considering their long half-life [87]. Although no studies have addressed their safety in pregnant patients, there is a theoretical risk of fetal growth retardation, increased blood pressure, and increase in fetal mortality, as seen in preclinical studies with CGRP antagonists [88]. However, it is worth noting that in non-human primates, administration of erenumab, a monoclonal antibody targeting the CGRP receptor, during pregnancy had no effects on pregnancy outcomes and postnatal growth [89].

In summary, in patients of childbearing potential, counseling regarding contraception, and the risks of the different antimigraine drugs during pregnancy is strongly advised, especially when prescribing medications such as antiepileptics. If patients decide to stop taking contraceptives, discussing the different acute and preventative treatment options is recommended to adjust treatment accordingly. It is also important to highlight to the patients that in the case of an unplanned pregnancy, they should inform their physician to readjust treatment [72, 90].

### Child and adolescent health considerations in headache medicine

Around 11% of the pediatric population suffers from migraine and 17% from tension-type headache [91]. Boys and girls have a similar one-year prevalence of migraine until puberty, after which the prevalence increases in both genders, with a more substantial rise observed in females than males [70, 92].

Similar to its impact on adults, migraine during childhood is linked to significant disability and considerable societal costs [93]. Children and adolescents with migraine experience impaired functioning in different areas of their lives, such as school, home, and social activities. This reduced functioning negatively affects their health-related quality of life and can influence their peer relationships [94]. The high prevalence of migraine among the younger population and its significant level of disability highlights the importance of clinical care to manage and prevent this condition within this age group effectively.

Accurately identifying migraine in pediatric patients can be challenging due to difficulties in describing symptoms, and differences in clinical features between childhood and adult migraines can complicate early diagnosis. Migraine attacks in children are typically shorter, lasting at least two hours, and the pain is generally milder than in adults, often affecting the bilateral frontotemporal regions [95]. The similarities between migraine and other headache disorders, like tension-type headache and sinusitis, can result in misdiagnoses. In a pediatric study, approximately 40% of children with migraines were initially misdiagnosed as having sinusitis [96].

While there have been advances in the study of treatments for migraine in pediatric and adolescent patients, the available treatment options remain somewhat limited. Many therapies are still based on data from a relatively small number of RCTs [93]. Approved therapies for acute attacks in children and adolescents with migraine include acetaminophen, NSAIDs like ibuprofen and naproxen, and triptans [97]. The best care model for migraine prevention should be interdisciplinary, incorporating self-management, lifestyle interventions, and tailored behavioral, nutraceutical, or pharmacological treatments. Due to the significant placebo effect and potential side effects of pharmacological therapies, non-pharmacological approaches are suggested as the first-line preventive treatment [98, 99]. Among pharmacological options, topiramate is the only one approved for this age group [94, 97]. Several RCTs are underway to evaluate the efficacy and safety of monoclonal antibodies targeting the CGRP pathway in children and adolescents with migraine [97]. These trials will pave the way for new therapies that could be available in the near future.

### Equity care access

Access to headache disorders care depends on the availability and approval of medications, the financial coverage of medical service, and the knowledge of general practitioners or headache specialist around the correct choice of care [100]. Due to poor public awareness of headache disorders, these are frequently neglected by policy makers or left undertreated by patients and healthcare providers [101].

Care for migraine and cluster headache shows access inequity worldwide. This has been further enhanced following the release of the novel disease-specific medications targeting CGRP. To begin with, Asian, African and Latin American countries have often been excluded from the clinical trials [102]. Further, while the USA and Europe have been seeing widespread approval and release of these treatments for migraine, most of them remain unavailable in most countries worldwide [103]. Below we look at some specific examples regarding the differences in availability and inequity of access for these novel drugs in two different scenarios.

#### Health inequity in migraine – the UK rimegepant refusal example

Management for migraine has greatly changed with the widespread use of the anti-CGRP monoclonal antibodies (erenumab, galcanezumab, fremanezumab, eptinezumab) and gepants (rimegepant, ubrogepant, atogepant). One of the crucial characteristics of gepants is that, unlike any other migraine drug, they can be used for both acute and preventive treatment, with rimegepant in particular representing both types of approaches (dual-use therapies) [102]. The drug, available at the dose of 75 mg in orally disintegrating form, was first tested for acute migraine relief in two large RCTs [104, 105]. Successively, a long-term open-label safety study allowed to record a reduction in migraine days per month with rimegepant taken every other day, prompting its testing for preventive treatment as well [106]. Rimegepant showed superiority to placebo on reduction of mean number of migraine days per month and has since been approved for acute and preventive use in Europe and the USA.

In February 2023, the UK National Institute for Health and Care Excellence (NICE) issued a statement not recommending rimegepant to be issued within the National Health Service for the acute treatment of migraine with or without aura in adults [107]. This decision was mostly due to uncertainty around cost-effectiveness of the treatment. Successively, NICE considered the acute and preventive recommendation separately and approved rimegepant for preventive treatment of episodic migraine in England [108].

The refusal of rimegepant use in the public health system in England was highly challenged by physicians and patient groups as it had the potential to increase health inequality among patients. In fact, migraine prevalence and incidence are known to present large disparities related to socioeconomic status and education levels, with marginalized communities being more exposed to underdiagnosis and lack of adequate treatment [109]. Not allowing for a novel treatment to be accessed equally by patients across the same country can lead to a worsening of those health disparities. In particular, it was argued that as rimegepant does not seem to cause any relevant vasoconstriction, it may represent a safer treatment option for patients who are not allowed triptans due to vascular comorbidities [67]. This could allow for a reduction in the migraine burden of patients who already have concomitant health problems, such as coronary artery disease, cerebrovascular disease, and systemic hypertension [110]. Further, as rimegepant can be used with high frequency for prevention, it is conceivable that it may not increase the risk of MOH. MOH represents in itself a significant burden that is also linked to disparity aspects such as geography, environment, ethnicity and culture, as well as quality and availability of medical care [111].

Finally, the decision taken by NICE also had the potential of influencing other government entities and public health institutes around the world, and its impact on the global burden of migraine could thus have extended well beyond the UK [112].

It is therefore excellent news that, on 18^th^ October 2023, NICE reverted this decision, and has now made rimegepant available as an option for the acute treatment of migraine with or without aura in adults. However, conditions require that at least 2 triptans have been tried without sufficient effect, or, if triptans are contraindicated or not tolerated, that NSAIDs and paracetamol have been tried without sufficient effect [113].

#### Health inequity in cluster headache

Cluster headache is a rare albeit highly disabling headache disorder (prevalence ~ 0.1%). Usual analgesics have little, if any, effect on cluster headache attacks. Acute and preventive treatment differ from that of other headache disorders, including migraine [114]. This makes the correct diagnosis a crucial step towards effective treatment. Worldwide, the diagnosis of cluster headache is often delayed for years [115, 116], and even more so in women [117] and adolescents [115]. This delay could be more pronounced in countries of the global south, where prevalent health issues like infectious diseases take precedence. Thus, inequity of cluster headache care starts with availability of physicians aware of the disorder and its treatment [100, 118].

Effective acute treatment is expensive, requiring either rapidly acting triptans (i.e., nasal or subcutaneous formulations) or high-flow oxygen [119]. Reimbursement issues are frequent [120]. Likely, there are parts of the world where these therapies are accessible only to wealthy patients. Some cluster headache patients have multiple attacks per day and consequently need triptans in a frequency that may exceed the recommended upper daily dosing limit, making reimbursement even more difficult. Oxygen is a highly effective acute therapy but needs highly developed logistics, making its availability at home difficult for large parts of the world [121]. Reimbursement of high-flow oxygen can also be a challenge and varies from country to country [120, 122]. Transitional treatment with corticosteroids may be more easily available, but needs close surveillance to avoid long-term use and its known adverse effects.

Cluster headache preventive therapy differs from that of other headache disorders. There are few high-quality RCTs, and medications are off label in many countries [119]. Verapamil is effective for many patients, but needs high doses, regular controls, and comes with cardiovascular risks. Therefore, initiation of this therapy needs a physician with a special expertise in headache.

There are other preventive therapies effective in some patients: lithium, that also requires close monitoring, and topiramate, which is often administered at doses higher than those for migraine. However, not all patients benefit, and there is a large unmet need for specific, highly effective, tolerable and easy-to-use preventive medication [123]. As CGRP plays an important role in cluster headache [124], assessing the effect of anti-CGRP therapies in cluster headache was a logical step. The only data currently published are those for galcanezumab, which at a 300 mg dose met the primary endpoint in episodic cluster headache [125] but not in chronic cluster headache [126]. On the basis of these data, the US Food and Drug Administration (FDA) approved galcanezumab for the treatment of episodic cluster headache in June 2019 [127]. Galcanezumab was also approved in other countries, such as the Republic of Korea. In contrast, the EMA refused approval in February of 2020, because evidence was considered too weak compared to possible risks [128]. Hence, galcanezumab at the 300 mg was not marketed in Europe and numerous other countries, including the Republic of Korea, despite receiving approval. Headache centers treating severely affected cluster headache patients have used galcanezumab (mostly 240 mg) in selected cases, with good results [129–131]. There are also some reports of patients with comorbid migraine and cluster headache, where treatment of migraine with an anti-CGRP antibody improved both migraine and cluster headache [132, 133]. In view of the high unmet need of cluster headache patients, associated with substantial suffering, and the limited evidence for existing preventive therapies, the EMA decision seems harsh, and creates substantial inequity [116].

### Future directions

The 2030 Agenda for Sustainable Development of the United Nations (ASD-2030) defines as one of its Sustainable Development Goals (SDG) to “Ensure healthy lives and promote well-being for all at all ages” [134]. In alignment with this SDG, addressing headache disorders on a global scale is imperative due to their high prevalence and disabling impact [134].

A recent consensus paper by international headache experts outlined specific proposals and actions for achieving this SDG in the field of headache disorders, which we endorse in the present review [134].

In the context of headache services, a key focus should be on providing targeted headache training at the primary level. Particularly in low- and middle-income countries, there is a need to promote neurology training, with a specific emphasis on headache management. This could be facilitated through collaborations with local academic institutions or mentorship programs [134]. In instances where a shortage of neurologists exists, non-specialized healthcare workers can be trained to recognize and treat common headache disorders. The incorporation of telemedicine can significantly enhance headache access, especially in rural areas, while expanding virtual learning opportunities can benefit healthcare professionals and foster cross-country knowledge exchange [134].

Implementing multi-modal disease management strategies must be context-specific, considering the socioeconomic and cultural setting, as well as the availability of medications [135, 136]. The overarching objective is to deliver evidence-based treatments tailored to individual patients, considering disease severity and characteristics, and adapting to the available and affordable medications in each specific country. This approach holds true for both acute and preventive medications, not only in low- and middle-income countries but also in high-income countries lacking an adequate number of headache experts [137–139].

Acknowledging that novel and potentially more effective medications may not be universally accessible, an initial step involves a more informed and widespread use of available evidence-based basic treatments [140–142]. Elevating disease awareness and enhancing education globally are pivotal steps in steering healthcare in the right direction.

## Conclusion

Disparities in healthcare, access to treatment and medication availability represent a real concern for headache medicine. As one of the most common and disabling disorders affecting individuals over their lifetime, headache management is particularly sensitive to differences in national health services. Further, its prevalence and severity are typically affected by gender, age and socioeconomic backgrounds, deepening the inequity of care in certain populations. These differences have been lately enhanced by the introduction of novel, albeit expensive, drugs that will likely revolutionize headache treatment in the coming years.

Healthcare professionals, headache experts and policy makers should be aware of these health disparities and strive to reduce the boundaries of access to headache management in the future, in order to allow equitable treatment of patients all over the world.

## Data Availability

Not applicable.

## Abbreviations

CGRP: Calcitonin gene-related peptide
EMA: European Medicines Agency
FDA: Food and Drug Administration
MOH: Medication overuse headache
NICE: National Institute for Health and Care Excellence
NSAIDs: Non-steroidal anti-inflammatory drugs
RCTs: Randomized controlled trials
WHO: World Health Organization
